# The possible molecular mechanisms of farnesol on the antifungal resistance of *C. albicans* biofilms: the regulation of *CYR1* and *PDE2*

**DOI:** 10.1186/s12866-018-1344-z

**Published:** 2018-12-04

**Authors:** Shengyan Chen, Jinping Xia, Chengxi Li, Lulu Zuo, Xin Wei

**Affiliations:** 1 Jiangsu Key Laboratory of Oral Diseases, Nanjing Medical University, Nanjing, 210029 China; 2 Department of Oral Medicine, Stomatology Hospital Affiliated to Nanjing Medical University, Nanjing, 210029 China; 3 Suzhou Hospital Affiliated to Nanjing Medical University, Suzhou Science and Technology Town Hospital, Suzhou, 215153 China

**Keywords:** *Candida albicans* biofilms, Resistance, Farnesol, *CYR1*, *PDE2*

## Abstract

**Background:**

Farnesol has potential antifungal activity against *Candida albicans* biofilms, but the molecular mechanism of this activity is still unclear. Farnesol inhibits hyphal growth by regulating the cyclic AMP (cAMP) signalling pathway in *C. albicans*, and *CYR1* and *PDE2* regulate a pair of enzymes that are directly responsible for cAMP synthesis and degradation. Here, we hypothesize that farnesol enhances the antifungal susceptibility of *C. albicans* biofilms by regulating *CYR1* and *PDE2*.

**Results:**

The resistance of the *CYR1*- and *PDE2*-overexpressing strains to caspofungin, itraconazole and terbinafine was increased in planktonic cells, and that to amphotericin B was increased in biofilms. Meanwhile, the biofilms of the *CYR1*- and *PDE2*-overexpressing strains were thicker (all *p* < 0.05) and consisted of more hyphae than that of the wild strain. The intracellular cAMP levels were higher in the biofilms of the *CYR1*-overexpressing strain than that in the biofilms of the wild strain (all *p* < 0.01), while no changes were found in the *PDE2*-overexpressing strain. Exogenous farnesol decreased the resistance of the *CYR1*- and *PDE2*-overexpressing strains to these four antifungals, repressed the hyphal and biofilm formation of the strains, and decreased the intracellular cAMP level in the biofilms (all *p* < 0.05) compared to the untreated controls. In addition, farnesol decreased the expression of the gene *CYR1* and the protein CYR1 in biofilms of the *CYR1*-overexpressing strain (all *p* < 0.05) but increased the expression of the gene *PDE2* and the protein PDE2 in biofilms of the *PDE2*-overexpressing strain (all *p* < 0.01).

**Conclusions:**

The results indicate that *CYR1* and *PDE2* regulate the resistance of *C. albicans* biofilms to antifungals. Farnesol suppresses the resistance of *C. albicans* biofilms to antifungals by regulating the expression of the gene *CYR1* and *PDE2*, while *PDE2* regulation was subordinate to *CYR1* regulation.

## Background

*Candida albicans*, existing as a commensal fungus in healthy individuals, is the major fungal pathogen in humans that can cause systemic candidiasis in immunocompromised patients, especially in organ transplant recipients, HIV-positive patients or those with autoimmune diseases [1]. In clinical infections of *C. albicans*, the fungi commonly form biofilms, which are associated with chronic infections. Biofilms are protected niches for microorganisms, where they are safer from antibiotic treatment than they are in their planktonic form, and can provide a source of persistent infection. Biofilm-related infections caused by *C. albicans* can become a major threat to public health, as these infections are refractory and result in a reservoir for continued infection [2–4]. Therefore, it is crucial to find the resistance mechanisms of *C. albicans* biofilms and to explore novel drugs and compounds that may enhance the efficacy of traditional antifungals in the therapeutic armamentarium against biofilm-related infections.

Farnesol, an exogenous chemical molecule, has a broad range of effects on *C. albicans*, but the most prominent of these are its ability to influence *C. albicans* morphology by inhibiting the yeast-to-hypha transition [5, 6] and its antifungal activities against *C. albicans* biofilms [7, 8]. Our previous research indicated that farnesol inhibited the development of *C. albicans* biofilms of resistant strains, and there were synergistic effects of farnesol in combination with antifungals [9, 10]. Further studies suggested that farnesol-dependent inhibition of hyphal formation involved the cAMP pathway [5, 11, 12]. Farnesol inhibited hyphal growth as an adenylate cyclase (CYR1) inhibitor and exerted a direct effect on intracellular cAMP levels [1, 11]. Another study found that farnesol upregulated *PDE2* expression in *C. albicans* biofilms grown for 24 h [13]. Those studies did not report any mechanism of the antifungal activities of farnesol on the *C. albicans* biofilms to be associated with *CYR1* or *PDE2*, and the mechanism needs to be explored.

The yeast-to-hypha transition is correlated with the antifungal resistance of *C. albicans.* As previously reported, cAMP is the key element in triggering hyphal formation [1, 14, 15]. CYR1 and PDE2 regulate a pair of enzymes that are directly responsible for cAMP synthesis and degradation, respectively [16]. The activation of cAMP signalling is due to the loss of cAMP phosphodiesterase, PDE2, or an increase in cAMP adenylate cyclase, CYR1 [17–19]. Further studies found that *CYR1* and *PDE2* were associated with the resistance of planktonic *C. albicans* to antifungals, and the mutants of *C. albicans* with a deletion of *CYR1* or *PDE2* had increased sensitivity to azole antifungals [20, 21]. However, the role of overexpressing *CYR1* and *PDE2* in the resistance of *C. albicans* biofilms and the role played by farnesol in this process need to be elucidated.

Therefore, we hypothesized that *CYR1* and *PDE2* regulate the resistance of *C. albicans* biofilms to antifungals and that farnesol increases the susceptibility of *C. albicans* biofilms to antifungals by regulating the gene expression of *CYR1* and *PDE2* in the cAMP pathway. The antifungal resistance of *C. albicans* biofilms formed by *CYR1*- and *PDE2*-overexpressing strains was examined in the presence or absence of farnesol and was compared to that in biofilms formed by the wild strain. Additionally, morphological changes were examined using CLSM and SEM. The intracellular cAMP level of *C. albicans* biofilms was detected by an ELISA. Furthermore, the expression of genes and proteins was analysed using RT-qPCR and western blotting, respectively.

## Results

### *CYR1* and *PDE2* are involved in the resistance of *C. albicans* to antifungals

**Fig. 1:**
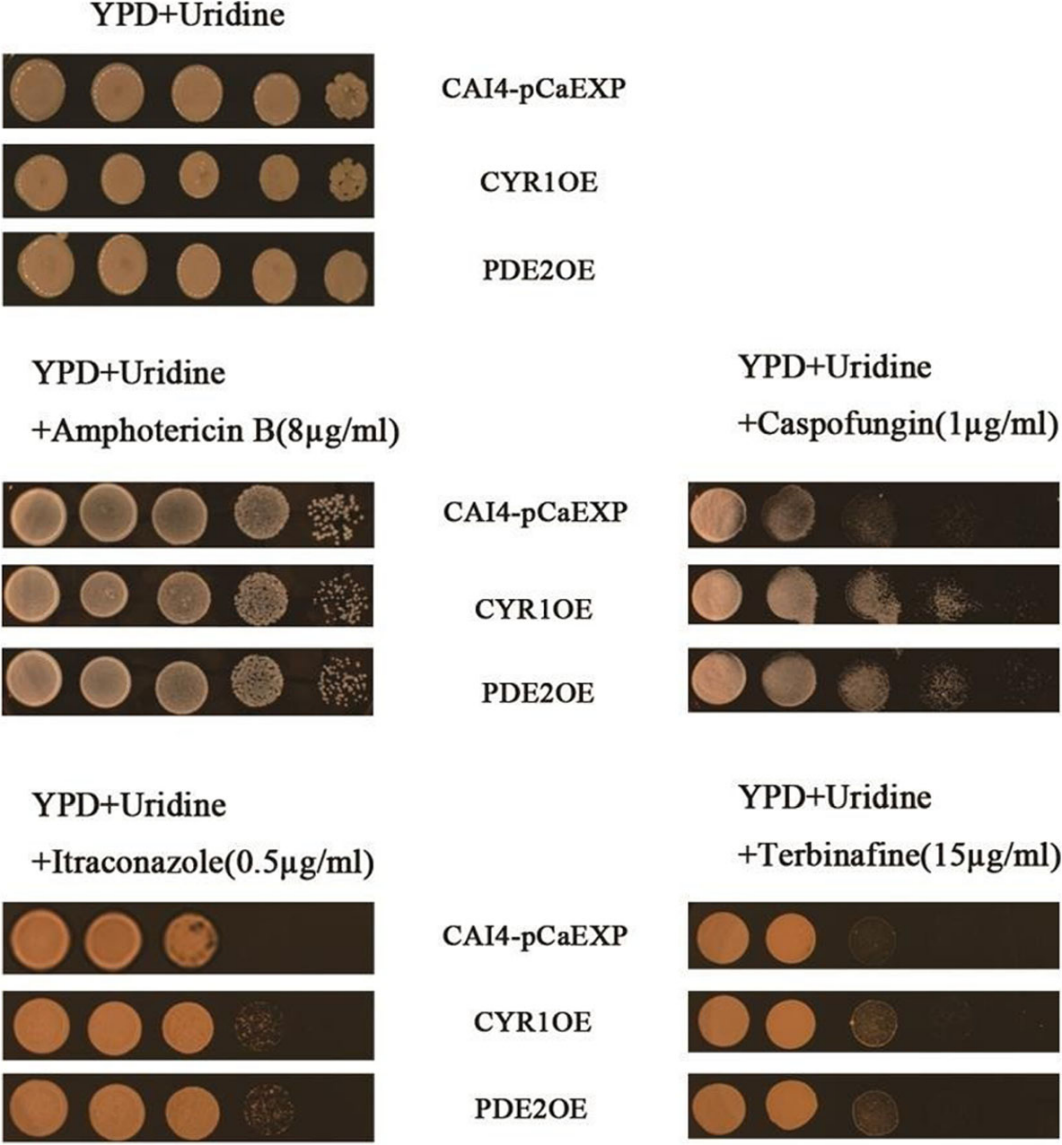
The susceptibility of *C. albicans* to antifungals. The concentrations of the drugs used above: Fluconazole: 4 μg/ml; Amphotericin B: 8 μg/ml; Caspofungin: 1 μg/ml; Itraconazole: 0.5 μg/ml; Terbinafine: 15 μg/ml. The planktonic form of *C. albicans* of the CYR1OE and PDE2OE strains was more resistant to caspofungin, itraconazole and terbinafine than was that of the wild strain

For the XTT assay, biofilms formed from the *CYR1*-overexpressing strain (CYR1OE) and the *PDE2*-overexpressing strain (PDE2OE) had higher values for the sessile minimum inhibitory concentration (SMIC) that caused a ≥ 50% reduction in the metabolic activity of the biofilms (SMIC_50_) of amphotericin B than did the wild strain (CAI4-pCaEXP) at all studied phases (Table 1). There were no significant differences in the SMIC_50_ values of caspofungin, itraconazole and terbinafine among the three strains (CYR1OE, PDE2OE and CAI4-pCaEXP). For the spot assay, the planktonic form of *C. albicans* of the CYR1OE and PDE2OE strains was more resistant to caspofungin, itraconazole and terbinafine than was that of the wild strain (Fig. 1).

**Table 1:** In vitro susceptibility of *C. albicans* biofilms to antifungals

Strains	SMIC_50_ of antifungals (μg/ml)			
	6 h	12 h	24 h	36 h
amphotericin B				
CAI4-pCaEXP	1	0.5	1	0.25
CYR1OE	4	8	> 16	16
PDE2OE	4	4	1	16
caspofungin				
CAI4-pCaEXP	0.125	> 8	> 8	> 8
CYR1OE	0.125	> 8	> 8	> 8
PDE2OE	8	> 8	> 8	> 8
itraconazole				
CAI4-pCaEXP	> 16	> 16	> 16	> 16
CYR1OE	> 16	> 16	> 16	> 16
PDE2OE	> 16	> 16	> 16	> 16
terbinafine				
CAI4-pCaEXP	> 256	> 256	> 256	> 256
CYR1OE	> 256	> 256	> 256	> 256
PDE2OE	> 256	> 256	> 256	> 256

### Farnesol increased the activities of antifungals against *C. albicans* biofilms of the CYR1OE and PDE2OE strains

The biofilms of the CYR1OE strain exposed to farnesol showed lower SMICs of amphotericin B (biofilm phase at 6, 12, 24 and 36 h) (Table 2), caspofungin (biofilm phase at 6, 12, 24 and 36 h) (Table 2), itraconazole (biofilm phase at 12 and 24 h) (Table 3) and terbinafine (biofilm phase at 6, 12 and 24 h) (Table 3) than did those that were not exposed to farnesol. Moreover, biofilms of the PDE2OE strain exposed to farnesol showed lower SMICs of amphotericin B (biofilm phase at 6, 12 and 36 h) (Table 2), caspofungin (biofilm phase at 6, 24 and 36 h) (Table 2), itraconazole (biofilm phase at 12, 24 and 36 h) (Table 3) and terbinafine (biofilm phase at 6, 12 and 24 h) (Table 3) than did those that were not exposed to farnesol. However, the farnesol-treated biofilms of the CYR1OE and PDE2OE strains showed higher SMICs of itraconazole (biofilm phase at 6 h) than those that were not treated with farnesol (Table 3).

**Table 2:** The SMICs of amphotericin B and caspofungin in *C. albicans* biofilms

Farnesol (μM)	amphotericin B(μg/ml)			caspofungin(μg/ml)		
	CAI4-pCaEXP	CYR1OE	PDE2OE	CAI4-pCaEXP	CYR1OE	PDE2OE
6 h						
0	1	4	4	0.125	0.125	8
50	1	4	4	0.125	0.125	8
100	0.5	2	4	0.0625	0.0625	8
200	1	4	2	0.0625	0.0625	0.625
300	1	4	1	0.0625	0.0625	0.25
600	1	4	1	0.0625	0.0625	0.25
1200	1	2	1	0.0625	0.125	0.125
12 h						
0	0.5	8	4	> 8	> 8	0.0625
50	0.5	4	4	> 8	8	0.0625
100	0.5	4	2	> 8	> 8	0.0625
200	0.5	4	2	0.25	8	0.0625
300	1	4	2	0.25	0.0625	0.0625
600	1	8	1	0.25	0.0625	0.0625
1200	16	8	2	0.0625	0.125	0.0625
24 h						
0	1	> 16	1	> 8	> 8	> 8
50	1	16	1	> 8	8	> 8
100	1	16	1	> 8	0.125	> 8
200	0.5	8	1	> 8	0.0625	0.25
300	1	8	1	> 8	0.0625	0.0625
600	0.125	8	1	2	0.0625	0.125
1200	0.125	8	1	> 8	0.0625	0.125
36 h						
0	0.25	16	16	> 8	> 8	> 8
50	0.25	16	16	4	1	> 8
100	2	8	8	1	0.5	> 8
200	0.125	4	4	0.25	0.5	0.125
300	0.125	4	4	0.125	0.5	0.125
600	0.25	4	2	0.0625	0.5	0.25
1200	0.125	4	2	4	0.5	0.5

**Table 3:** The SMICs of itraconazole and terbinafine in *C. albicans* biofilms

Farnesol (μM)	itraconazole(μg/ml)			terbinafine(μg/ml)		
	CAI4-pCaEXP	CYR1OE	PDE2OE	CAI4-pCaEXP	CYR1OE	PDE2OE
6 h						
0	> 16	0.0625	0.3125	> 256	> 256	> 256
50	> 16	0.0625	0.0625	> 256	> 256	> 256
100	> 16	0.125	0.0625	> 256	> 256	> 256
200	> 16	0.125	> 16	> 256	2	4
300	> 16	0.125	> 16	> 256	2	2
600	> 16	0.125	> 16	> 256	4	8
1200	> 16	0.125	> 16	> 256	16	4
12 h						
0	> 16	> 16	> 16	> 256	> 256	> 256
50	> 16	> 16	> 16	> 256	> 256	> 256
100	> 16	> 16	> 16	> 256	> 256	> 256
200	> 16	0.5	0.125	> 256	> 256	> 256
300	> 16	0.5	0.125	> 256	2	4
600	> 16	0.5	0.125	> 256	2	4
1200	1	1	1	> 256	4	32
24 h						
0	> 16	> 16	> 16	> 256	> 256	> 256
50	> 16	> 16	> 16	> 256	> 256	> 256
100	> 16	> 16	> 16	> 256	> 256	> 256
200	> 16	16	2	> 256	1	1
300	> 16	0.25	0.125	> 256	2	2
600	> 16	0.25	0.25	> 256	> 256	> 256
1200	> 16	0.5	0.5	> 256	> 256	> 256
36 h						
0	> 16	> 16	> 16	> 256	> 256	> 256
50	> 16	> 16	> 16	> 256	> 256	> 256
100	> 16	> 16	> 16	> 256	> 256	> 256
200	0.125	> 16	> 16	> 256	> 256	> 256
300	0.5	> 16	16	> 256	> 256	> 256
600	1	> 16	2	> 256	> 256	> 256
1200	2	> 16	4	> 256	> 256	> 256

Under treatment with the same concentration of farnesol, the biofilms of the CYR1OE strain showed higher SMICs of amphotericin B (biofilm phase at 6, 12, 24 and 36 h) (Table 2) and SMICs of itraconazole (biofilm phase at 36 h) (Table 3) than did those of the wild strain (CAI4-pCaEXP). Further, the biofilms of the PDE2OE strain showed higher SMICs of amphotericin B (biofilm phase at 6, 12 and 36 h) (Table 2), caspofungin (biofilm phase at 6 h) (Table 2) and itraconazole (biofilm phase at 36 h) (Table 3) than did those of the wild strain. In addition, compared to the biofilms of the PDE2OE strain, the biofilms of the CYR1OE strain showed higher SMICs of amphotericin B (biofilm phase at 6, 12, 24 and 36 h) (Table 2) under treatment with the same concentration of farnesol.

### Farnesol changed the morphology of *C. albicans* biofilms formed form the CYR1OE and PDE2OE strains

**Fig. 2:**
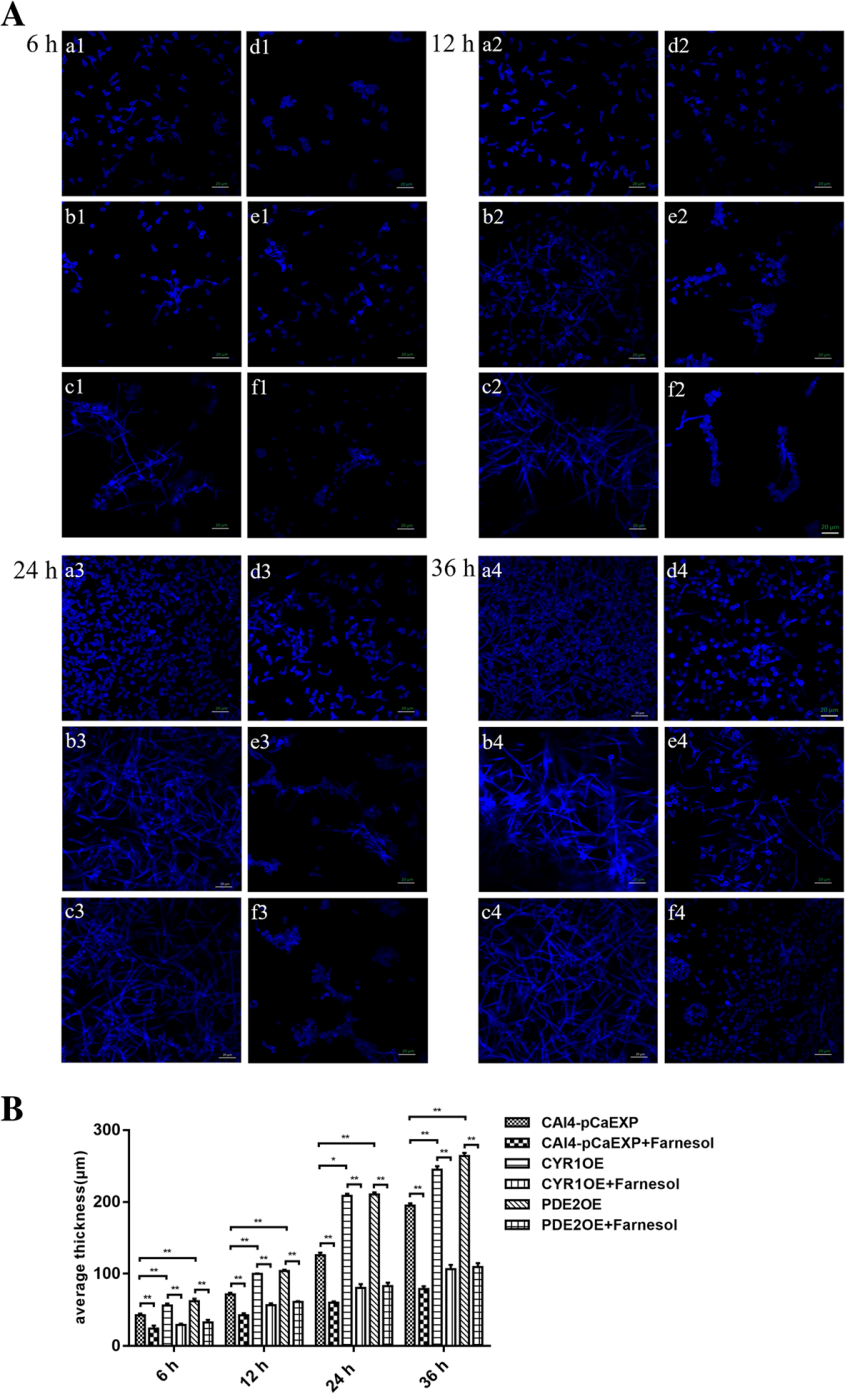
Confocal Laser Scanning Microscopy (CLSM) images of *C. albicans* biofilms formed from the CYR1OE and PDE2OE strains. **A:** a1–4: Farnesol untreated wild strain. b1–4: Farnesol untreated CYR1OE strain. c1–4: Farnesol untreated PDE2OE strain. d1–4: Farnesol treated wild strain. e1–4: Farnesol treated CYR1OE strain. f1–4: Farnesol treated PDE2OE strain. Scale bars represent 20 μm for 400× magnifications. The biofilms of d1–4, e1–4 and f1–4 consisted of fewer hyphae, but more pseudohyphae and yeast than did those of a1–4, b1–4 and c1–4. Additionally, the biofilms of b1–4 and c1–4 consisted of more hyphae and pseudohyphae than did that of a1–4. **B:** The biofilms exposed to farnesol were thinner than that without farnesol and the biofilms of the CYR1OE and PDE2OE strains were much thicker than that of the wild strain. Significance was calculated using one-way ANOVA with post ad-hoc Dunnett’s multiple comparison test. *: *p* < 0.05. **: *p* < 0.01

**Fig. 3:**
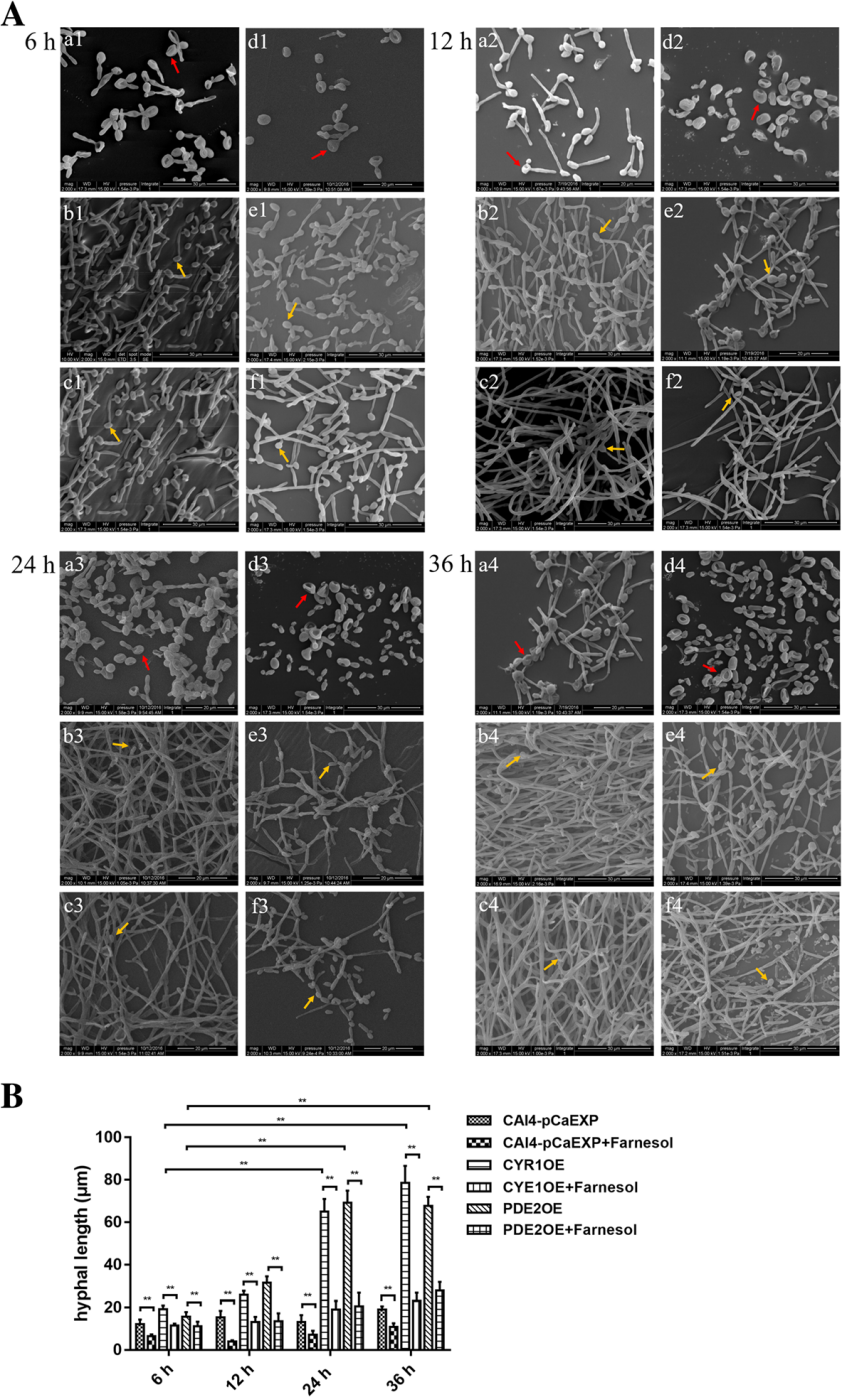
Scanning Electron Microscopy (SEM) images of *C. albicans* biofilms formed from the CYR1OE and PDE2 strains. **A:** a1–4: Farnesol untreated wild strain. b1–4: Farnesol untreated CYR1OE strain. c1–4: Farnesol untreated PDE2OE strain. d1–4: Farnesol treated wild strain. e1–4: Farnesol treated CYR1OE strain. f1–4: Farnesol treated PDE2OE strain. Magnification: 2000×. The surface of the cells in a1–4, d1–4 was uneven (red arrows), while the surface of cells in b1–4, c1–4, e1–4, f1–4 was smooth with spores (yellow arrows). The other results were the same as Fig. 2A. **B:** The biofilms formed from the CYR1OE and PDE2OE strains exposed to farnesol consisted of shorter hyphae than that without farnesol. The hyphae extended much longer in the later phases (24 and 36 h) of the biofilms than did that in the early phases (6 h)**.** Significance was calculated using one-way ANOVA with post ad-hoc Dunnett’s multiple comparison test. *: *p* < 0.05. **: *p* < 0.01

The results of CLSM and SEM all showed that the biofilms of the farnesol-treated groups consisted of fewer hyphae but more pseudohyphae and yeast than did the controls without farnesol treatment (Figs. 2A and 3A). Moreover, the biofilms of CYR1OE and PDE2OE strains consisted of more hyphae and pseudohyphae than did those of the wild strain (Figs. 2A and 3A). Furthermore, SEM showed that the cell surfaces in the biofilms of the wild strain were uneven (Fig. 3A a1–4 and d1–4), while the cell surfaces in the biofilms of the CYR1OE and PDE2OE strains were smooth with spores (Fig. 3A b1–4, c1–4, e1–4 and f1–4). No obvious difference was observed between the cell surfaces of the farnesol-treated biofilms and the untreated controls. The quantitative assessment for the CLSM images showed that the biofilms exposed to farnesol were thinner than that without farnesol (all *p* < 0.01). The biofilms of the CYR1OE and PDE2OE strains were much thicker than that of the wild strain (all *p* < 0.05) (Fig. 2B). What’s more, the quantitative assessment for the SEM images showed that the biofilms of the CYR1OE and PDE2OE strains exposed to farnesol consisted of shorter hyphae than that without farnesol (all *p* < 0.01). The hyphae extended much longer in the later phases of the biofilms than did that in the early phases (all *p* < 0.01) (Fig. 3B)**.**

### Farnesol decreased the cAMP levels in the *C. albicans* biofilms of the CYR1OE and PDE2OE strains

**Fig. 4:**
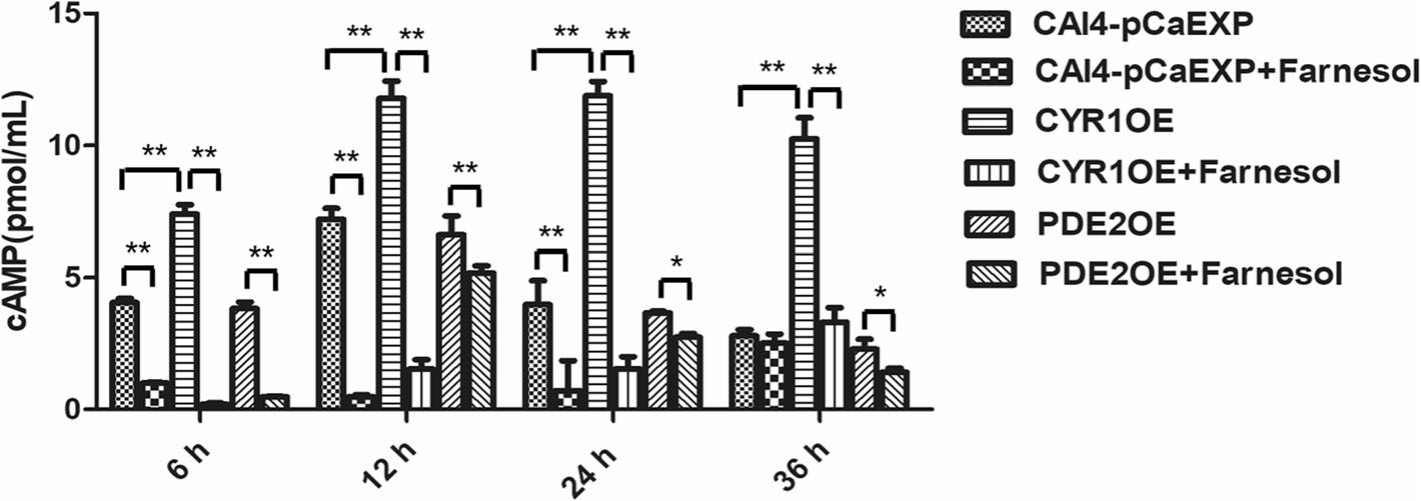
The level of cAMP from *C. albicans* biofilms in the presence of farnesol. Farnesol decreased the intracellular cAMP levels in the biofilms of the CYR1OE strain, the PDE2OE strain, and the wild strain compared to those of the respective untreated controls. In addition, compared to the cAMP levels in the wild strain, the cAMP levels in the CYR1OE strain were significantly higher in all studied phases. One-way analysis of variance (ANOVA) was employed to assess the statistical significance of differences in matched groups, while paired t-tests were performed for intra-group comparisons. *: *p* < 0.05. **: *p* < 0.01

The results of the ELISA showed that farnesol decreased the intracellular cAMP levels in the biofilms of the CYR1OE strain (all *p* < 0.01), the PDE2OE strain (all *p* < 0.05), and the wild strain (*p* < 0.01) (Fig. 4), compared to those of the respective untreated controls. In addition, compared to the cAMP levels in the wild strain, the cAMP levels in the CYR1OE strain were significantly higher in all studied phases (all *p* < 0.01), while those of the PDE2OE strain did not change in any studied phase (all *p* > 0.05) (Fig. 4).

### Farnesol decreased CYR1 expression but increased PDE2 expression

**Fig. 5:**
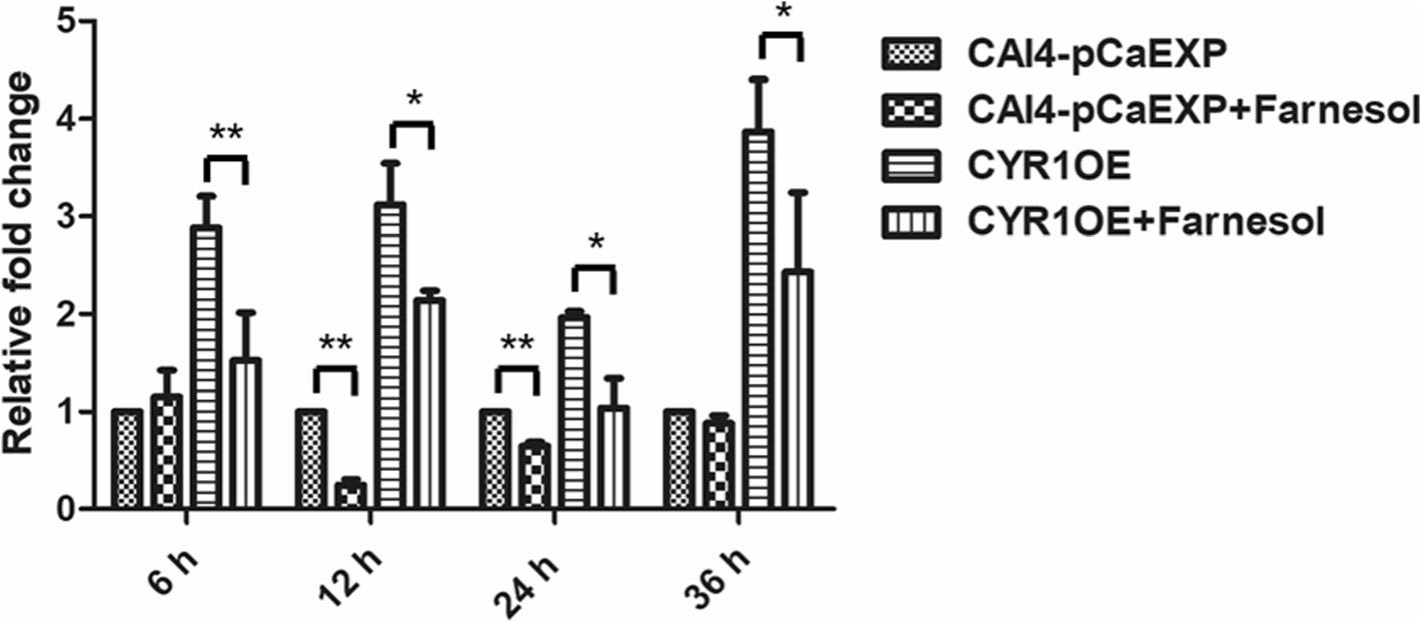
The expression of gene *CYR1* in the presence of farnesol. Farnesol decreased the expression of *CYR1* in the biofilms of the CAI4-pCaEXP strain at 12 and 24 h biofilm phases and decreased the expression of *CYR1* in the biofilms of the CYR1OE strain at 6, 12, 24 and 36 h biofilm phases compared to the respective untreated controls. One-way analysis of variance (ANOVA) was employed to assess the statistical significance of differences in matched groups, while paired t-tests were performed for intra-group comparisons. *: *p* < 0.05. **: *p* < 0.01

**Fig. 6:**
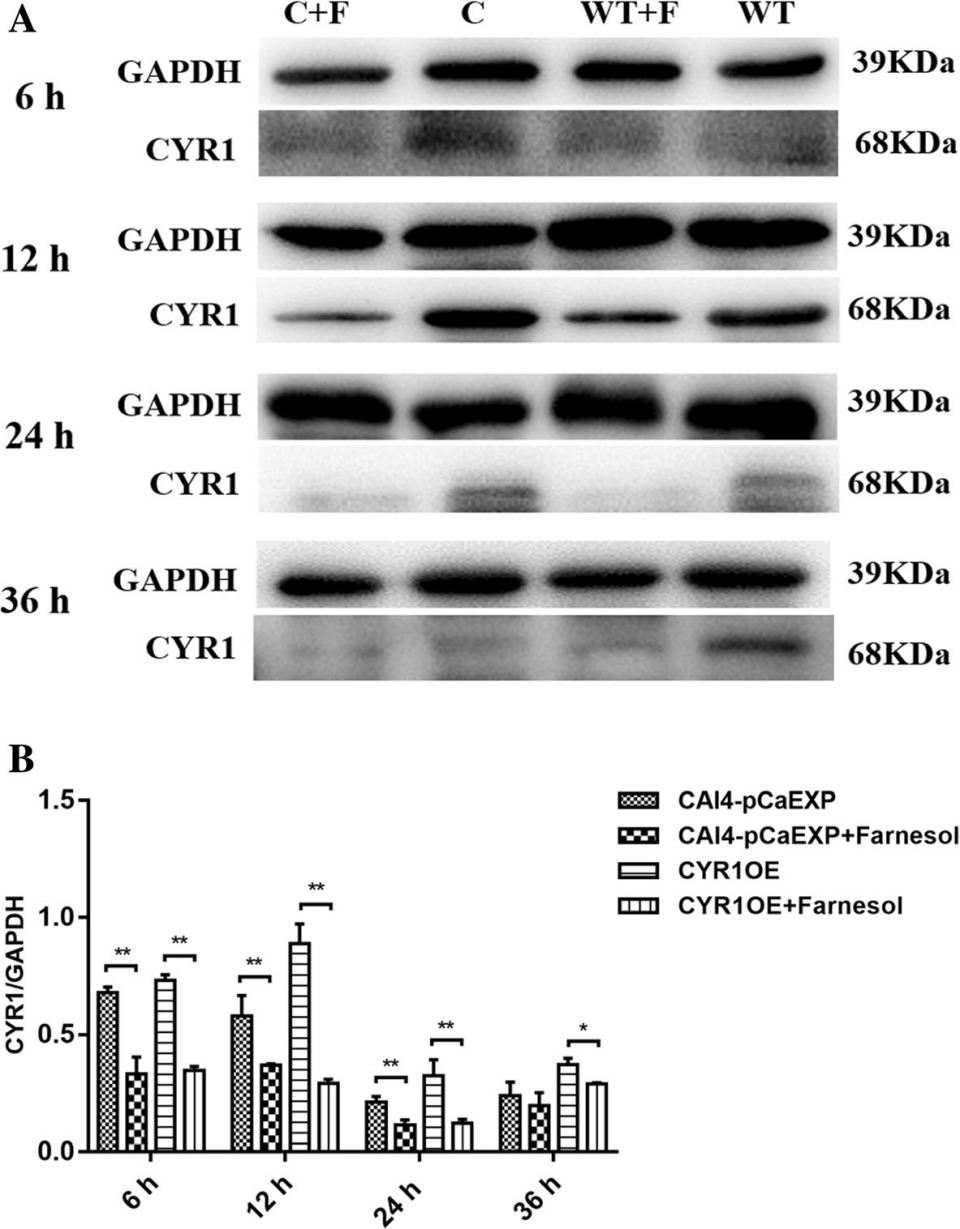
The expression of CYR1 protein in *C. albicans* biofilms. **A:** WT: Farnesol untreated wild strain (CAI4-pCaEXP); WT + F: Farnesol treated wild strain (CAI4-pCaEXP); C: Farnesol untreated *CYR1*-overexpressing strain (CYR1OE); C + F: Farnesol treated *CYR1*-overexpressing strain (CYR1OE). **a** and **b:** Farnesol decreased the expression of CYR1 of the CAI4-pCaEXP strain at 12, 24 and 36 h biofilm phases and decreased the expression of CYR1 of the CYR1OE strain at 6, 12 and 24 h biofilm phases compared to the respective untreated controls. Shown are means ± standard deviation of three independent experiments performed in duplicate. One-way analysis of variance (ANOVA) was employed to assess the statistical significance of differences in matched groups, while paired t-tests were performed for intra-group comparisons. *: *p* < 0.05. **: *p* < 0.01

**Fig. 7:**
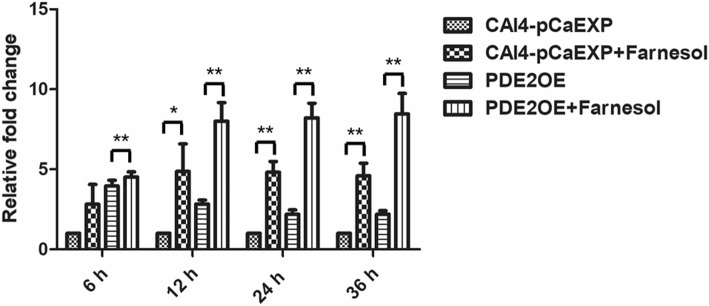
The expression of gene *PDE2* in the presence of farnesol. Farnesol increased the expression of *PDE2* in the biofilms of the CAI4-pCaEXP strain at 12, 24 and 36 h biofilm phases and increased the expression of *PDE2* in the biofilms of the PDE2OE strain at 6, 12, 24 and 36 h biofilm phases compared to the respective untreated controls. One-way analysis of variance (ANOVA) was employed to assess the statistical significance of differences in matched groups, while paired t-tests were performed for intra-group comparisons. *: *p* < 0.05. **: *p* < 0.01

**Fig. 8:**
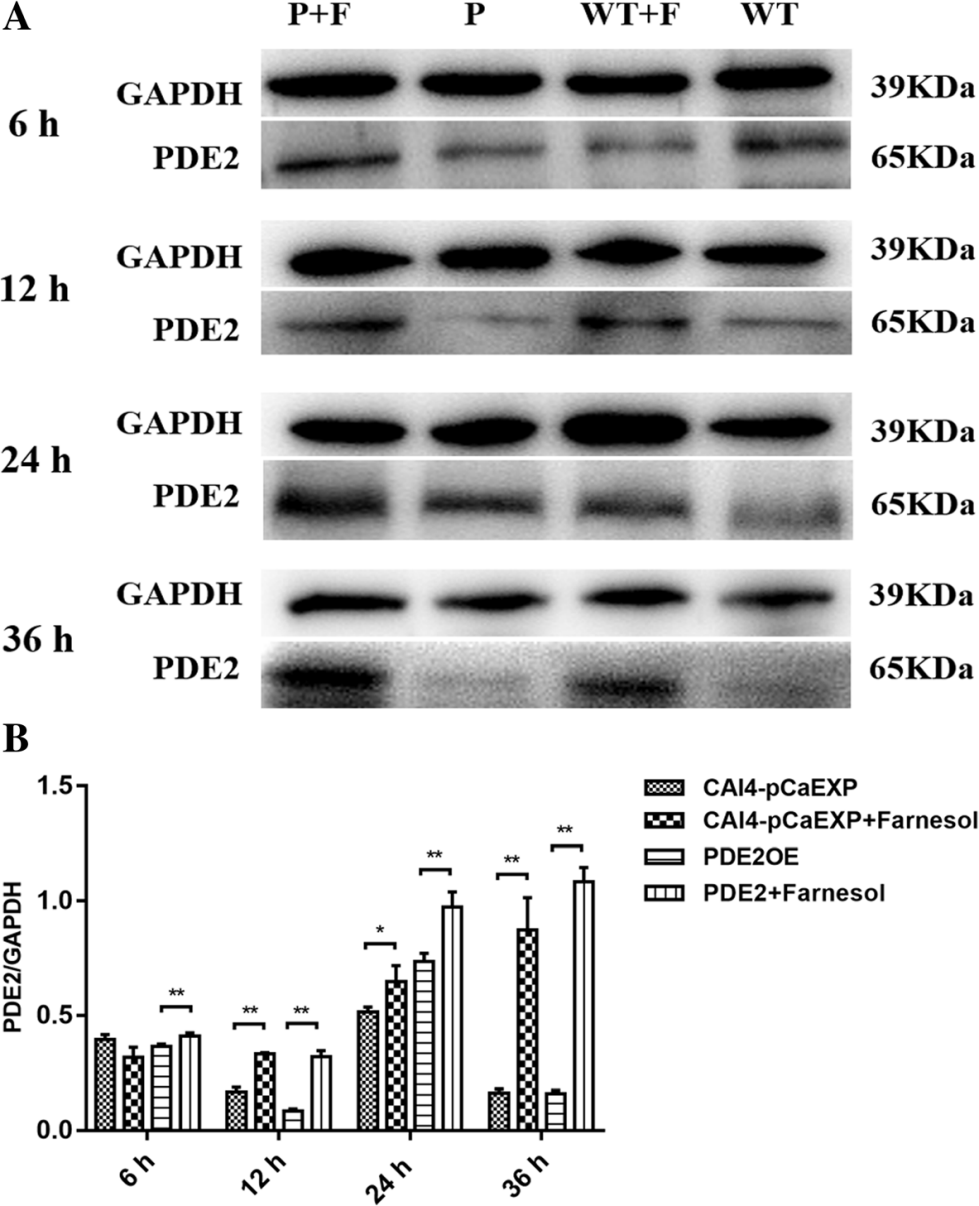
The expression of PDE2 protein in *C. albicans* biofilms. **a:** WT: Farnesol untreated wild strain (CAI4-pCaEXP); WT + F: Farnesol treated wild strain (CAI4-pCaEXP); P: Farnesol untreated *PDE2*-overexpressing strain (PDE2OE); P + F: Farnesol treated *PDE2*-overexpressing strain (PDE2OE). **a** and **b:** Farnesol increased the expression of PDE2 of the CAI4-pCaEXP strain at 12, 24 and 36 h biofilm phases and increased the expression of PDE2 of the PDE2OE strain at 6, 12, 24 and 36 h biofilm phases compared to the respective untreated controls. Shown are means ± standard deviation of three independent experiments performed in duplicate. One-way analysis of variance (ANOVA) was employed to assess the statistical significance of differences in matched groups, while paired t-tests were performed for intra-group comparisons. *: *p* < 0.05. **: *p* < 0.01

The results of RT-qPCR and western blotting showed that farnesol decreased the expression of the gene *CYR1* and the protein CYR1 in the biofilms formed from the wild strain (*p* < 0.01) and the CYR1OE strain (all *p* < 0.05), compared to the respective untreated controls (Figs. 5 and 6). Farnesol increased the expression of the gene *PDE2* and the protein PDE2 in the biofilms formed from the wild strain (*p* < 0.05) and the PDE2OE strain (all *p* < 0.01), compared to the respective controls (Figs. 7 and 8).

## Discussion

The cAMP pathway regulates the morphological interconversions in *C. albicans* biofilms and is determined by the processes of synthesis and hydrolysis [16]: adenylyl cyclases (CYR1) catalyse the conversion of ATP to cAMP, while phosphodiesterases (PDE1 and PDE2) hydrolyse cAMP to AMP [22]. The *C. albicans CYR1* regulated three developmental programmes, namely, invasive filamentous growth, phenotypic switch, and biofilm formation, while the deletion of *CYR1* prevented the growth of hyphae, and block the formation of mature biofilms [23–25]. In addition, previous studies also provided evidence that a *C. albicans* adenylate cyclase mutant (homozygous deletion) was hyper-susceptible to various antifungals, such as fluconazole, itraconazole, miconazole, terbinafine, fenpropimorph, amphotericin B, and caspofungin [21, 26]. In this study, the data revealed that a *CYR1*-overexpressing strain had a higher cAMP level and more hyphal growth and biofilm formation than did the wild strain. *CYR1* overexpression also increased the resistance of planktonic *C. albicans* to caspofungin, itraconazole and terbinafine and of sessile *C. albicans* to amphotericin B. The results confirmed that *CYR1* contributed to the resistance and formation of the biofilms, which suggested that *CYR1* is associated with the susceptibility of *C. albicans* biofilms to antifungals.

PDE2, a high-affinity phosphodiesterase, was required for hyphal development and cell wall integrity in *C. albicans* [16, 27]. The deletion of *PDE2* causes elevation of cAMP levels, prohibits normal hyphal development in hypha-inducing liquid medium and the biofilms development [27, 28]. In this study, the *PDE2*-overexpressing strain had increased hyphal growth and biofilm formation, which is consistent with the finding of a previous study by Jung WH et al. [28] that normal hyphal growth was prohibited in a *PDE2*-deletion strain. Moreover, a previous study showed that a strain with a *PDE2* deletion had decreased resistance of planktonic cells to amphotericin B, flucytosine and fluconazole [20]. Our data further found that *PDE2* overexpression increased the resistance of planktonic *C. albicans* to most antifungals, namely, caspofungin, itraconazole and terbinafine, and of sessile *C. albicans* to amphotericin B, which suggested that *PDE2* is associated with the resistance of *C. albicans* biofilms to antifungals.

Previous studies showed that farnesol specifically affected the activity of the cAMP signalling pathway and was able to bind to the cyclase domain of the adenylyl cyclase CYR1, exerting a direct effect on intracellular cAMP levels [1, 11, 12]. This study confirmed that farnesol directly inhibited CYR1 activity and decreased the level of intracellular cAMP and hypha formation in the biofilms of the *CYR1*-overexpressing and wild strains. In addition, farnesol decreased the SMICs of amphotericin B, caspofungin, itraconazole and terbinafine in *C. albicans* biofilms formed from the *CYR1*-overexpressing and wild strains. Further, farnesol decreased the expression of the gene *CYR1* and the protein CYR1 in those biofilms. The results suggested that farnesol decreased the antifungal resistance of *C. albicans* by reducing the expression of *CYR1*.

The study also found that farnesol inhibited hypha formation and decreased the level of intracellular cAMP in the *PDE2*-overexpressing and wild strains. In addition, farnesol decreased the SMICs of amphotericin B, caspofungin, itraconazole and terbinafine in the biofilms formed from the same strains. In addition, farnesol increased the expression of *PDE2* in the biofilm formed from those strains. The results suggested the overexpression of *PDE2* regulated by farnesol would not change the inhibitory effect of farnesol on the hyphal growth or the antifungal resistance in the biofilms. Farnesol regulation could decrease the expression of an upstream gene, *CYR1*, which encodes adenylate cyclase and reduces cAMP level, and even if farnesol increases the expression of a downstream gene, *PDE2*, and enhances the phosphodiesterase level, there is not enough cAMP to be degraded by the extra phosphodiesterase. The results suggested that *PDE2* regulation was subordinate to *CYR1* regulation by farnesol. Consistent with this finding, a previous study indicated that the high-affinity cAMP phosphodiesterase PDE2 was not required for repression of hypha formation by farnesol [11]. These data suggested that *PDE2* might have a functionality auxiliary to the antifungal specificity of farnesol, which needs to be explored in the future.

It has been generally accepted that hyphal development in *C. albicans* requires elevated cAMP levels. Intracellular cAMP levels increased during the yeast-to-hypha transition, and the maximum level coincided with maximum germ tube formation [29]. This study showed that the overexpression of *CYR1* increases the cAMP levels and the hypha formation of the biofilms, while the overexpression of *PDE2* increases the hypha formation without changing the cAMP levels. Jung WH et al. found that Pde2p-catalysed cAMP hydrolysis is required for normal growth and development of hyphae in *C. albicans* [28], and the deletion of *PDE2* causes elevated cAMP levels, severe growth defects and greatly reduced levels of the transcription of an important hypha-associated gene, *EFG1* [16, 30]. The results were in accordance with those of a previous study, which suggested that cAMP level regulation by *PDE2* may be subordinate to that by *CYR1, EFG1* or other downstream genes associated with hyphal growth in *C. albicans* biofilms.

*C. albicans* biofilm formation proceeds in three distinct developmental phases: early (0 to 11 h), intermediate (12 to 30 h), and maturation (30 to 72 h) [31]. Farnesol, which can regulate the formation of *C. albicans* biofilms, decreased the resistance of biofilms grown for 12 h, 24 h and 36 h from *CYR1*-overexpressing and *PDE2*-overexpressing strains to itraconazole, while it increased the resistance of biofilms grown for 6 h to itraconazole. The results suggested that phase-specific mechanisms might be involved in the resistance of the biofilms.

## Conclusion

In summary, our study demonstrated that *CYR1* and *PDE2* are associated with the resistance of *C. albicans* biofilms to antifungals, and farnesol decreased the antifungal resistance of *C. albicans* biofilms by regulating the expression of the gene *CYR1* and *PDE2*, while *PDE2* regulation by farnesol was subordinate to *CYR1* regulation by farnesol.

## Methods

### Strains and media

The strains and plasmids used in this study were provided by the State Key Laboratory of Pharmacy Genetic Engineering, Second Military Medical University, Shanghai, China and are listed in Table 4. The CYR1OE and PDE2OE strains were transformed from CAI4 using *CYR1*-pCaEXP and *PDE2*-pCaEXP plasmids, respectively [32–34]. The wild strain CAI4-pCaEXP was transformed from CAI4 using the pCaEXP plasmid. A rich (yeast extract peptone dextrose (YPD) + uridine) (2% peptone, 1% yeast extract, 2% glucose and 0.01% uridine) medium was prepared for wild strain incubation, and a selective (SD-ura-met-cys) medium was prepared for CYR1OE and PDE2OE incubation.

**Table 4:** Strains and plasmids used in these studies

	Genotype and Description	reference
*C. albicans* strains		
CAI4	ura3:: λimm434/ura3:: λimm434	Fonzi and Irwin (1993)
	URA3 auxotrophic strain	
CAI4-pCaEXP	ura3:: λimm434/ura3:: λimm434-(pCaEXP URA3)	In this study
	Wild strain transformed with pCaEXP used as a control of overexpression experiment	
CYR1OE	ura3:: λimm434/ura3:: λimm434-(*CYR1*-pCaEXP *CYR1, URA3*)	In this study
	*CYR1*-overexpressing strain	
PDE2OE	ura3:: λimm434/ura3:: λimm434-(*PDE2*-pCaEXP *PDE2, URA3*)	In this study
	*PDE2*-overexpressing strain	
Plasmids		
pCaEXP	URA3 and MET3 promoter integrating plasmid	R. S. Care et al. (1999)
*CYR1-*pCaEXP	Constructed by integration of *CYR1*	In this study
*PDE2-*pCaEXP	Constructed by integration of *PDE2*	In this study

### Biofilm formation

The strains were grown overnight in 10 ml of YPD + uridine medium at 30 °C in a roller drum [35]. Then, the cells were harvested and diluted into RPMI-1640 medium (Gibco Ltd., Paisley, UK) at an initial concentration of 1 × 10^6^ cells/ml. The suspensions were inoculated into the polystyrene surface of 96-well plates or culture discs (Corning Inc., N.Y., USA) [31]. After 2 h, non-adherent cells were removed by washing twice with sterile phosphate-buffered saline (PBS). The plates were then incubated for 6, 12, 24, or 36 h for the development of biofilms. The media were replenished once every 2 h, and farnesol (Sigma Chemical Co., St. Louis, MO) was added subsequently, depending on the experimental groups.

### Susceptibility tests for *C. albicans*

For the XTT reduction assay for sessile *C. albicans*, stock solutions of amphotericin B (2.5 mg/ml), caspofungin (10 mg/ml), itraconazole (20 mg/ml) (Sigma-Aldrich, St Louis, MO, USA) and terbinafine (50 mg/ml) (Selleckchem, Houston, TX, USA) were prepared in sterile distilled water (for amphotericin B) or dimethyl sulfoxide (DMSO; for caspofungin, itraconazole and terbinafine). Farnesol was dissolved in 100% (vol/vol) methanol. According to the method M27-A3 from the Clinical and Laboratory Standards Institute (CLSI) (2008), drugs were prepared in serial 2-fold dilutions, and their final concentrations ranged from 0.125 to 16 μg/ml for amphotericin B, 0.0625 to 8 μg/ml for caspofungin, 0.03125 to 16 μg/ml for itraconazole and 0.5 to 256 μg/ml for terbinafine. After incubation with the antifungal agents for 24 h, biofilm SMICs were determined using the XTT (0.25 mg/ml) and menadione (25 μM) assays [36]. The SMIC_50_ was determined as the minimum antifungal concentrations that caused a ≥ 50% reduction in the metabolic activity of the biofilms compared to that of the control. The tests were repeated in triplicate for each assay, and each group was tested in triplicate on different days.

For the spot assay of planktonic *C. albicans* [37], the strains were incubated in YPD + uridine medium and grown for 16 h in an orbital shaker at 30 °C. Antifungals were added to the medium at a concentration of 8 μg/ml for amphotericin B, 1 μg/ml for caspofungin, 0.5 μg/ml for itraconazole and 15 μg/ml for terbinafine. For the spot assay, 5 μl of five-fold serial dilutions of yeast suspensions (cells suspended in normal saline to an OD_600_ nm of 0.1) was spotted onto YPD + uridine plates in the absence or presence of the antifungal drugs [34]. Growth was not affected by the presence of solvent used in the examination. The assay was repeated three times and growth differences were measured after incubation at 30 °C for 24–48 h.

### Morphological observation by CLSM and SEM

Biofilms were washed with PBS and fixed with 4% paraformaldehyde. For CLSM observation, biofilms were stained with 500 μl calcofluor white stain [38] (0.0025 g/ml; Sigma Chemical Co. St. Louis, MO) for 30 min at 37 °C in the dark. Then, the biofilms were observed and images were taken using a Zeiss LSM700 microscope (Carl Zeiss, Inc., Oberkochen, Germany) with a 495 nm argon ion laser. The three-dimensional image analysis software ZEN (Carl Zeiss, Inc., Oberkochen, Germany) was used to analyse the thickness of biofilms (depending on the height of the biofilm) formed from different groups. For SEM analysis, biofilms were placed in 2.5% (vol/vol) glutaraldehyde overnight at 4 °C. Samples were dehydrated in a series of washes with increasing concentrations of ethanol (70% for 10 min, 95% for 10 min and 100% for 20 min), dried in a desiccator and coated with gold powder [35, 39]; then, the biofilms were observed using a scanning electron microscope (1530VP, LEO, Oberkochen, Germany). The hyphal length of each germinated spore was measured using the software Image J (1.51, National Institutes of Health, Bethesda, MD, USA). All assays were performed in triplicate on three different occasions to ensure reproducibility and representative images were taken.

### Assay of intracellular cAMP

The levels of intracellular cAMP of the biofilms were examined using an ELISA according to the method of Yun et al., with slight modifications [23]. Briefly, biofilms were washed once with water and once with MES buffer (10 mM MES containing 0.1 mM EDTA; pH = 6) and then dried in a desiccator and supplemented with MES buffer with 10% trichloroacetic acid up to the same concentration. The cells were frozen in liquid nitrogen and thawed on ice twice and sonicated under chilled conditions (twice at 130 W for 2 min) using an ultrasonic cell crusher (UH-500B, Celton, Tianjin, China). After centrifugation, trichloroacetic acid was extracted four times with water-saturated ether. The cAMP content was measured with a cAMP enzyme immunoassay system (Amersham, Sigma, USA) following the manufacturer’s instructions. The tests were repeated in triplicate for each assay, and each group was tested in triplicate on different days.

### RT-qPCR

Isolation of the total RNA of *C. albicans* biofilms was carried out using a modified hot phenol method as described previously [40]. Reverse transcription was performed on 1 mg of total RNA using a cDNA synthesis kit (TaKaRa Bio Co., Ltd., Dalian, China) as specified in the manufacturer’s instructions, and the synthesized cDNA was stored at − 80 °C until needed. The synthetic cDNA described above was used for real-time PCR analysis, which was performed on an ABI 7300 Fast Real-time PCR machine (Applied Biosystems, Rotkreuz, Switzerland) using Absolute qPCR SYBR Green Mix (Thermo Scientific, Waltham, MA, USA). Amplification was achieved using the following cycle settings: 10 min at 95 °C followed by 40 cycles of 95 °C for 15 s, 55 °C for 60 s, and 72 °C for 20 s. After the amplification, a melting curve was analysed to ensure the absence of primer dimers. Expression of genes was calculated using the 2^-ΔΔCt^ method [10]. *ACT1* was used as a reference gene. The primers were all designed by Shanghai Generay BioTech Co., Ltd. (Table 5). The test was repeated three times.

**Table 5:** Primer sequences used in this study

Primer name	Sequence (5′ → 3′)
*CYR1*-F	AAGACGATGAAACAGCCACA
*CYR1*-R	AGTTGGTAAGCCAGTAGTCGG
*PDE2*-F	ATGCTGTGGGACATTGGAGT
*PDE2*-R	TAAAGTAGTGCCGTTGGTGC
*ACT1*-F	GCCGGTGACGACGCTCCAAGAGCTG
*ACT1*-R	CCGTGTTCAATTGGGTATCTCAAGGTC

### Western blotting

Total protein extracts were prepared from an immunoprecipitation protocol as previously described [41]. Protein samples were mixed with a one-fifth volume of 5 × sample buffer for SDS-PAGE. Samples were boiled for 5 min and separated by SDS-PAGE using an 8–10% acrylamide gel. Proteins were electro-transferred to PVDF membranes (Bio-Rad Laboratories, Inc.) and blocked with 5% skimmed milk in PBS with 0.1% Tween-20. Blots were hybridized with antibodies against *C. albicans* CYR1 or PDE2 (1:1000), followed by incubation in PBST containing the secondary antibody (1:10000 dilution) for 1 h. GAPDH (1:1000 dilution, Bioworld, Minneapolis, MN, USA) was used as a reference protein. Protein bands were detected by using a chemiluminescence system (Merck & Co., Inc., Kenilworth, NJ, USA). All operations were repeated three times and the gray value were measured with Gel-Pro Analyzer Software (Media Cybernetics, Rockville, MD, USA). The CYR1 and PDE2 level was standardized by the gray value ratio of CYR1/GAPDH and PDE2/GAPDH, respectively.

### Statistical analysis

One-way analysis of variance (ANOVA) was employed to assess the statistical significance of differences in matched groups, while paired t-tests were performed for intra-group comparisons. Differences were considered statistically significant at *p* < 0.05 or *p* < 0.01. The analyses were performed with SPSS statistics 17.0 software (SPSS Inc., Chicago, IL).

## Abbreviations

cAMP: cyclic AMP
cDNA: complementary DNA
CLSI: Clinical and Laboratory Standards Institute
CLSM: confocal laser scanning electron microscopy
CYR1OE: *CYR1*-overexpressing
DMSO: dimethyl sulfoxide
ELISA: enzyme-linked immunosorbent assay
MES: morpholineethanesulfonic acid
PBS: phosphate-buffered saline
PBST: phosphate-buffered saline with Tween-20
PDE2OE: *PDE2*-overexpressing
PVDF membrane: microporous membrane of polyvinylidene fluoride
RT-qPCR: reverse transcription quantitative real-time polymerase chain reaction
SEM: scanning electron microscopy
SMIC: sessile minimum inhibitory concentration
XTT: 2,3-bis(2-methoxy-4-nitro-5-sulfophenyl)-5-[(phenylamino)carbonyl]-2H-tetrazolium hydroxide
YPD: yeast extract peptone dextrose
